# Aortic valve stenosis and osteoporosis: insights from a mouse model

**DOI:** 10.1186/s12872-025-05037-4

**Published:** 2025-07-31

**Authors:** Hannah Billig, Johanna Schmitt, Lamia Singer, Christoph Bourauel, Frank A. Schildberg, Werner Masson, Nikola Lübbering, Wenzel Vogel, Sven Perner, Marta Stei, Sven Niepmann, Miriam Silaschi, Farhad Bakhtiary, Georg Nickenig, Sebastian Zimmer

**Affiliations:** 1 Department of Cardiology, University Hospital Bonn, Venusberg-Campus 1, 53127 Bonn, Germany; 2 Oral Technology, University Hospital Bonn, 53111 Bonn, Germany; 3 Orthodontic Department, University Hospital Bonn, 53111 Bonn, Germany; 4 Department of Orthopedics and Trauma Surgery, University Hospital Bonn, 53127 Bonn, Germany; 5 Institute of Pathology, University of Luebeck, 23562 Luebeck, Germany; 6 Department of Heart Surgery, University Hospital Bonn, 53127 Bonn, Germany

**Keywords:** Aortic valve stenosis, Animal model, Osteoporosis, Heart valve disease

## Abstract

**Background:**

Aortic valve stenosis (AVS) is the most common heart valve disease requiring intervention. Osteoporosis, affecting ~ 10% of those over 50, is linked to aortic valve calcification and increased AVS risk. However, its direct role in AVS development or progression remains unclear. Using a mouse model, we examined whether estrogen-deficiency-induced osteoporosis modifies early hemodynamic and histological remodeling of the aortic valve after mechanical injury, and whether zoledronic acid (ZA) treatment alters these processes.

**Methods:**

Osteoporosis was induced in mice via bilateral ovariectomy (OVX), with sham-operated controls. To prevent bone loss, ZA or vehicle were administered weekly via intraperitoneal injection. Two weeks post-OVX, AVS was induced by mechanically injuring the aortic valve under echocardiographic guidance, with control procedures (CTR) in separate groups. After model validation, C57BL/6J mice were assigned to six groups: WI Sham Vehicle, WI OVX Vehicle, WI OVX ZA, WI Sham ZA, CTR OVX Vehicle, and CTR OVX ZA. Echocardiography was performed at baseline and 2, 4, and 6 weeks post-injury. Bone density was assessed via micro-CT and histology, with peak aortic velocity as the primary endpoint. Mice were euthanized at 8 weeks for tissue analysis.

**Results:**

OVX induced a significant reduction in trabecular bone mineral density (TBM, 50.6%, *p* = 0.0025). Treatment with ZA effectively reversed bone resorption in OVX mice (*p* < 0.0001) and even enhanced trabecular structures compared to sham-operated animals (increase of TBM by 130%, *p* < 0.0001). Mechanical injury to the aortic valve successfully induced AVS, as evidenced by increased peak velocity (1294 vs. 2157 mm/s, *p* < 0.0001) and mean pressure gradient 2 weeks post-procedure (1.58 vs. 4.19 mmHg, *p* < 0.0001). However, neither OVX nor ZA treatment influenced the severity of AVS. Histological analyses confirmed aortic valve thickening following injury. Picrosirius red and CD68 staining revealed no differences in collagen content or immune cell infiltration of the aortic valve between osteoporotic and non-osteoporotic animals.

**Conclusion:**

OVX-induced osteoporosis did not affect AVS severity after mechanical injury in our study. This suggests osteoporosis may not directly influence AVS in the early, pre-calcific stage that was studied in this model. However, to overcome limitations of the study, further studies with longer durations or refined models are needed to confirm these findings.

**Supplementary Information:**

The online version contains supplementary material available at 10.1186/s12872-025-05037-4.

## Introduction

AVS is a progressive disease of the elderly, marked by the narrowing of the aortic valve, leading to significant morbidity and mortality if left untreated. The pathophysiology of AVS is predominantly driven by chronic inflammation, fibrosis and calcification processes [1–3]. Progressive cusp calcification closely recapitulates skeletal bone formation [4–7]. Epidemiologically, low bone-mineral density correlates with advanced calcific AVS, yet its role in pre-calcific remodeling is unknown. We therefore combined ovariectomy-induced osteoporosis with wire-injury AVS in mice to test whether bone loss– and its reversal with zoledronic acid (ZA)– modulates early fibrotic thickening and hemodynamic obstruction before calcification appears.

Central to this bone–valve axis is the receptor activator of nuclear κB (RANKL)/osteoprotegerin pathway: RANKL induces osteogenic differentiation of valvular interstitial cells, yet stimulates osteoclast-mediated bone resorption [5, 8]. Additionally, bone morphogenetic proteins, matrix Gla protein, vitamin D, and vitamin K represent key shared mechanisms underlying both valvular calcification and bone turnover [6, 9]. Similarly, while inflammatory cytokines and modified lipoproteins drive mineralization in vascular and valvular cells, they stimulate osteoclast activation in the bone [5, 10, 11].

Bone loss is a widespread health concern, affecting 43.9% of the population aged over 50 years, with osteoporosis observed in 10.3% [12].

The relationship between bone health and cardiovascular diseases has become a critical area of research, particularly given the aging population and the increasing prevalence of these conditions [9, 13–17].

Several cohorts report that lower bone-mineral density correlates with a higher risk of AVS– termed the “calcification paradox” [18–22]. Evidence is varied, however, likely owing to heterogeneous study designs, small samples, and residual confounding [23].

Although osteoporosis is more prevalent in women, the manifestation of AVS differs markedly between the sexes: women typically develop a predominantly fibrotic form of AVS, whereas men are far more likely to exhibit pronounced calcification of the valve cusps [24, 25]. This sex-specific pattern underscores the complexity of the bone-valve axis and highlights the need for studies specifically designed to unravel the mechanistic links among bone metabolism, valve fibrosis, and calcification.

## Methods

### Animals

All procedures involving animals were conducted in strict accordance with institutional guidelines and the regulations of the German animal protection law (LANUV North Rhine-Westphalia; file number 81-02.04.2022.A042).

Exclusion criteria for animals and data points were defined a priori to ensure consistency and minimize bias. To monitor animal well-being, we utilized an individual animal documentation system, where health parameters such as body weight, general appearance, behavior, and surgical wound condition were recorded regularly. Additionally, a predefined Score Sheet was employed to systematically evaluate and quantify animal welfare. This Score Sheet included criteria such as weight loss, activity levels, grooming behavior, and other signs of discomfort or distress. Animals exceeding humane endpoints as defined in the Score Sheet were excluded from the study and euthanized according to institutional guidelines.

Group allocation was performed by a researcher not involved in the conduct of the experiment or outcome assessment to minimize allocation bias. During the conduct of the experiment, the individuals performing the surgical procedures (e.g., ovariectomy, sham operation, and aortic valve injury) and administering treatments (ZA or vehicle) were aware of the group allocation to ensure proper execution of the protocol.

The animals were housed in a facility maintained at 22 °C, under a 12-hour light/dark cycle in cages of 5 animals. Both chow and drinking water were provided ad libitum.

To evaluate the model of bilateral OVX on bone density and quantify the effects of ZA, female C57BL/6J (wild type) mice were purchased from Charles River Laboratories, Wilmington, MA, and randomly distributed among the experimental cohorts (6 mice for OVX + vehicle, 6 mice for sham procedure + vehicle, and 6 mice for each dose of OVX + ZA). OVX was conducted at 12 weeks of age.

To evaluate the impact of osteoporosis on WI-induced AVS, 180 female C57BL/6J mice, were purchased from Charles River Laboratories, Wilmington, MA, and randomly distributed among the experimental cohorts. The cohort size for our animal experiments was determined based on a power analysis to ensure statistically significant results with adequate sensitivity. Preliminary data and published studies [26] were used to estimate expected effect sizes and standard deviations. Our aim was to achieve a power of 80%, identifying the minimum number of animals per group required to detect meaningful differences. Considering periprocedural mortality, we identified sizes of 30 animals per group.

Published studies and preliminary data show that peak aortic velocities after wire injury plateau by week 6 suggesting that the major hemodynamic impact of remodeling occurs early [27]. Because estrogen-deficient mice develop chronic osteoporotic pain, the follow-up was initially limited to 8 weeks to adhere to 3R principles while capturing the window of early, pre-calcific valve remodeling [28].

At baseline, spontaneous aortic valve stenosis with peak velocity of > 2000 mm/s was observed in 5 mice that was concomitantly excluded from the analysis. 9 mice died related to wire injury procedure (peri-procedural mortality of 5%). 166 mice remained for further investigation.

Mice were randomly distributed among experimental groups: WI Sham Vehicle, WI OVX Vehicle, WI OVX ZA, WI Sham ZA, CTR OVX Vehicle, and CTR OVX ZA.

### Ovariectomy

OVX was conducted at 12 weeks of age. Preoperative analgesia was administered subcutaneously (buprenorphine, 0.1 mg/kg) 30 min prior to anesthesia induction in an isoflurane chamber (3–5% isoflurane/1l O2/min), maintained at 1–2% isoflurane via a nose cone. Body temperature was regulated with an external heating pad. Eye protection was ensured with ophthalmic ointment.

The surgical site was prepared by shaving and extensive disinfection. A dorsal midline incision (ca. 0.5 cm) was made, followed by a small abdominal incision paravertebral. The ovary and associated adipose tissue were externalized, ligated, and excised. The procedure was performed on both sides for bilateral ovariectomy. Following organ removal, the incisions were closed with surgical sutures (RESORBA, Silk 6/0). Recovery took place in heated cages, with food and water provided at ground level. Postoperative analgesic therapy was provided for the first three days.

Successful OVX was confirmed by uterus weight at the end of experimentation (Supp. Mat. a).

### Wire injury

Induction of aortic valve injury or sham procedure was conducted two weeks after OVX. Experiments were performed as previously described [26]. Anesthesia was administered via intraperitoneal injection of xylazine hydrochloride (16 mg/kg) and ketamine hydrochloride (100 mg/kg), with body temperature maintained on a heating pad throughout the surgery. To prevent drying, eye ointment was applied.

A right paramedian cervical incision was made. The common carotid artery, as well as the internal and external carotid arteries, were exposed. A coronary wire (ASAHI INTEC, 0.36 mm diameter) bent at a 15° angle was introduced into the artery under echocardiographic guidance and advanced to the aortic valve level. Correct wire placement was verified via echocardiography in the parasternal long axis. The wire was manipulated to induce endothelial damage to the valve and standardized valve injury was achieved by moving it slightly in and out (10 times) and rotating it in place (100 times). The control group underwent a sham operation procedure, which involved the introduction of the wire into the vessel without causing injury to the valve.

Following the procedure, the right carotid artery was ligated, and the incision was closed with surgical sutures. Recovery took place in heated cages, with food and water provided at ground level. Postoperative analgesic therapy was provided for the first three days.

### Intraperitoneal injection of zoledronic acid

Zoledronic acid was administered intraperitoneally weekly for 8 weeks, beginning at the day of OVX/sham procedure. To minimize tissue irritation from repeated intraperitoneal injections, alternating injection sites in the lower right quadrant were used. Prior to the main experiments, a dose-response analysis was performed (2.5, 25, and 100 µg ZA/week, equivalent to approx. 0.1, 1, and 4 mg/kg, respectively). This analysis demonstrated that trabecular bone mineral density (BMD) already reached a plateau at the lowest dose (2.5 µg/week), with no further increases in BMD or adverse effects at higher doses (s. Supp. Mat. c). These findings align with previous reports showing that ZA’s bone-stabilizing effects saturate at low concentrations [29, 30]. Despite this saturation, we selected the highest dose (100 µg/week) for the main study to ensure robust suppression of bone turnover and enable potential effects on cardiovascular osteoclast-like cells throughout the experiment.

### Echocardiography

Cardiac function was evaluated using a Fujifilm Visualsonics Vevo 2100 Ultra High Frequency Imaging Platform. Echocardiography was performed as previously described [26]. Mice were anesthetized with 1.5% isoflurane, while their electrocardiogram, respiratory rate, and body temperature were continuously monitored. Prior to imaging, the fur on the chest was removed using a chemical depilatory agent to improve image clarity. Aortic valve peak velocity was assessed in the suprasternal view using a pulse-wave Doppler with angle correction between 40° and 50°. Examinations were conducted at baseline, and biweekly after aortic valve injury procedure by two partly blinded examiners.

### Euthanasia

Following the observation period of 8 weeks after ovariectomy, the animals were placed under general anesthesia using xylazine hydrochloride (16 mg/kg) and ketamine hydrochloride (100 mg/kg), administered intraperitoneally.

Adequate depth of anesthesia was confirmed by the absence of the interdigital reflex. Animals were then euthanized via exsanguination from the caudal vena cava while under deep anesthesia, ensuring they remained unconscious throughout the procedure. This method was selected to minimize animal suffering and was performed in full compliance with German animal welfare regulations, with approval from the local authority (LANUV, North Rhine-Westphalia, file number 81-02.04.2022.A042).

### µ-Computed tomography and quantitative image analysis

For the scanning procedure, femurs were carefully positioned and fixed vertically in a tube sample holder to ensure stable and symmetrical placement. The sample holder was securely affixed with silicone to the scanning tray to prevent any movements that could potentially affect high-resolution scanning accuracy. The femur samples were scanned using a SkyScan1172 µCT (Skyscan 1.6.1.5, 1174V2, Bruker, Massachusetts, USA) operating at 50 kV and 800 µA. A pixel size of 7.7 μm was utilized, with a rotation step of 0.2°, frame averaging set to 2, and a complete 360° trajectory. No aluminum or copper filters were employed during the scans, and each scanning session lasted approximately 2.5 h. For 3D volume reconstruction, NRecon software (Skyscan, v.1.6.1) was employed. Reconstruction parameters optimized for bone scans were applied, including a smoothing factor of 0, ring artifact reduction of 8, beam-hardening correction of 38%, and misalignment compensation tailored to the profile window for each specific case. The resulting reconstructions were saved as bit Bitmap files.

The regions of interest for bone analysis were located below the epiphyseal plate. NRecon reconstruction software (Ver.1.7, Bruker) and CTAn (CT-Analyser, Ver.1.18, Bruker) were used for three-dimensional image rebuilding and quantitative analysis. All data were calibrated against a standardized phantom specimen prior to further processing. The phantom used was a Bruker-µCT bone mineral density calibration rod, composed of epoxy resin with embedded fine calcium hydroxyapatite powder. The rod had a diameter of 4 millimeters and was made of two parts, each containing a different calcium hydroxyapatite concentration: one with 0.25 g/cm^2^ and the other with 0.75 g/cm^2^. This phantom was selected because its size and composition closely matched the cross-sectional area of the murine femur, ensuring accurate bone mineral density measurements. The small diameter of the rod helped minimize beam hardening effects, providing precise attenuation coefficient readings that are essential for reliable measurements in small bone samples, such as those from mice.

### Histological analysis

Hearts were flushed with 0.9% sodium chloride, embedded in tissue freezing medium, and sectioned at 8 μm thickness. A representative number of aortic valve sections were stained, with a minimum of 10 per group.

Aortic valve sections underwent hematoxylin and eosin staining following standard protocols. Tissue calcium and collagen deposition were respectively assessed using Alizarin Red S (Lifeline Cell Technology, Frederick, MD) and Pico-Sirius Red Staining (Sigma-Aldrich, Germany). Imaging was performed with light/polarization microscopy (Sirius Red) at 10x magnification (Axio Observer, Zeiss, Germany), and semi-automatically analyzed using Zeiss ZEN Imaging Software.

The approximate region of each aortic valve cusp was manually selected. The outline of the cusp was marked by the software, reviewed by the investigator, and recorded as an area. A schematic illustration can be found in Supp. Mat. b.

Femurs were placed in paraformaldehyde, embedded in paraffin and sectioned at 4 μm thickness without decalcification. Sections underwent hematoxylin and eosin staining following standard protocols.

### Immunofluorescence

Macrophage infiltration was quantified via CD68 staining. Sections were fixed in acetone, washed in phosphate-buffered saline, and blocked with 1% bovine serum albumin. Incubation with primary antibody anti-CD68 rat IgG2a (Acris Antibodies, Germany) was performed overnight at 4 °C in a humid chamber protected from light. The following day, sections were washed and incubated with a secondary antibody (Cy3 AffiniPure Donkey anti-Rat IgG, Jackson ImmunoResearch Laboratories Inc). After washing, nuclei were counterstained with Vectashield mounting medium containing DAPI (Vector Laboratories). Images were captured with an Axio Observer (Zeiss, Germany).

Aortic valve calcification was assessed by Osteosense™ 680EX staining (PerkinElmer, USA). Cryosections stored at -80 °C were allowed to dry at room temperature and were fixed in 4% formaldehyde for 10 min. After rinsing with tap water for 5 min, sections were washed twice with 1× phosphate-buffered saline (PBS) for 5 min each. The tissue sections were dried and encircled with a hydrophobic pen. OsteoSense™ 680EX solution (1:200 in PBS) was freshly prepared and applied (50 µL per section). Incubation was performed overnight at 4 °C in a humid chamber protected from light. The following day, sections were and counterstained with Vectashield mounting medium containing DAPI (Vector Laboratories). Imaging was performed using an Axio Observer (Zeiss, Germany).

### Statistical analysis

All statistical analyses were conducted using GraphPad Prism (version 10, GraphPad Software, San Diego, CA, USA). Data normality was assessed using the Shapiro-Wilk test prior to analysis. As the majority of data followed a normal distribution, parametric tests were employed for consistency. One- or two-way-analysis of variance (ANOVA) was used to assess significance, with the Geisser-Greenhouse correction applied to address potential violations of sphericity. For multiple comparisons, post-hoc analysis was performed using the Tukey correction to adjust for potential Type I errors.

All results are reported as mean and standard deviation. A p value of less than 0.05 was considered statistically significant.

Ejection fraction velocity ratio was calculated as LVEF/4 × (peak jet velocity in m/s)^2^, as described before [31].

## Results

### Zoledronic acid counteracts ovariectomy-induced osteoporosis

**Fig. 1:**
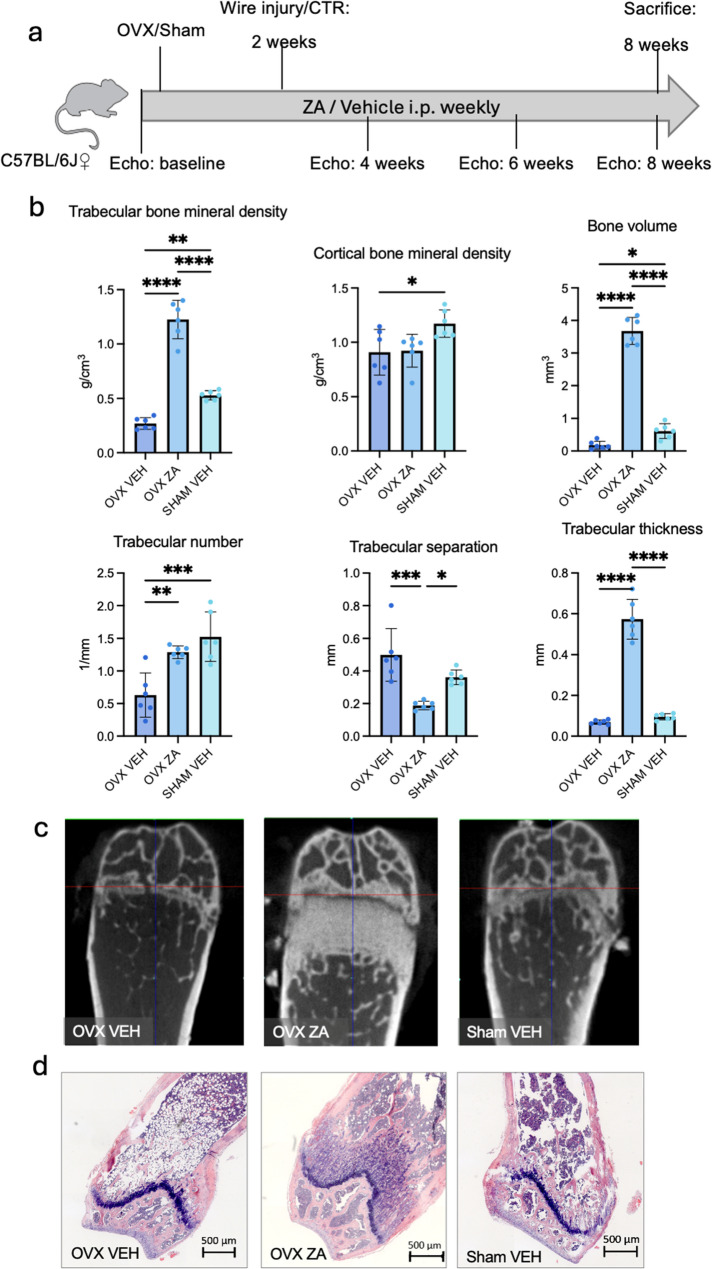
Experimental timeline and bone analysis after ovariectomy (OVX) and zoledronic acid (ZA) treatment. **a** Timeline of interventions: OVX or sham surgery, wire injury (WI), and weekly ZA/vehicle (VEH) injections. **b** µCT analysis shows OVX-induced reductions in trabecular and cortical bone parameters, which ZA treatment significantly preserved. **c** Representative µCT images of distal femurs highlight structural differences between groups. **d** Histological (H&E) images confirm ZA’s protective effects on trabecular bone

Bilateral ovariectomy (OVX) was used to induce osteoporosis in 12-week old female C57BL/6J mice. Compared to sham-operated animals, OVX led to a significant reduction in trabecular and cortical bone mineral density (reduction of trabecular BMD by 50.6%, *p* = 0.0025; reduction of cortical BMD by 22%, *p* = 0.04), bone volume (BV, by 77.6%, *p* = 0.045) and trabecular numbers (Tb.N., reduction by 61%, *p* = 0.0003; increase in trabecular separation, Tb.Sp., by 24.7%, *p* = 0.04) after 8 weeks as shown by µ-computed tomography analysis and histological images (Fig. 1b, d).

Weekly injections of 100 µg of the bisphosphonate ZA were used to prevent osteoporosis. ZA led to increased trabecular BMD in comparison to OVX (by 354%, *p* < 0.0001) and sham groups (by 130%, *p* < 0.0001), but had no effect on cortical BMD. ZA led to an increase in trabecular structures in comparison to OVX (increase in BV *p* < 0.0001, Tb.N. *p* = 0.0045, trabecular thickness *p* < 0.0001, decrease of Tb.Sp. *p* = 0.0002) and sham operated animals (increase in BV *p* < 0.0001, trabecular thickness *p* < 0.0001, decrease of Tb.Sp. *p* = 0.02).

### Wire injury procedure leads to hemodynamically relevant aortic valve stenosis

**Fig. 2:**
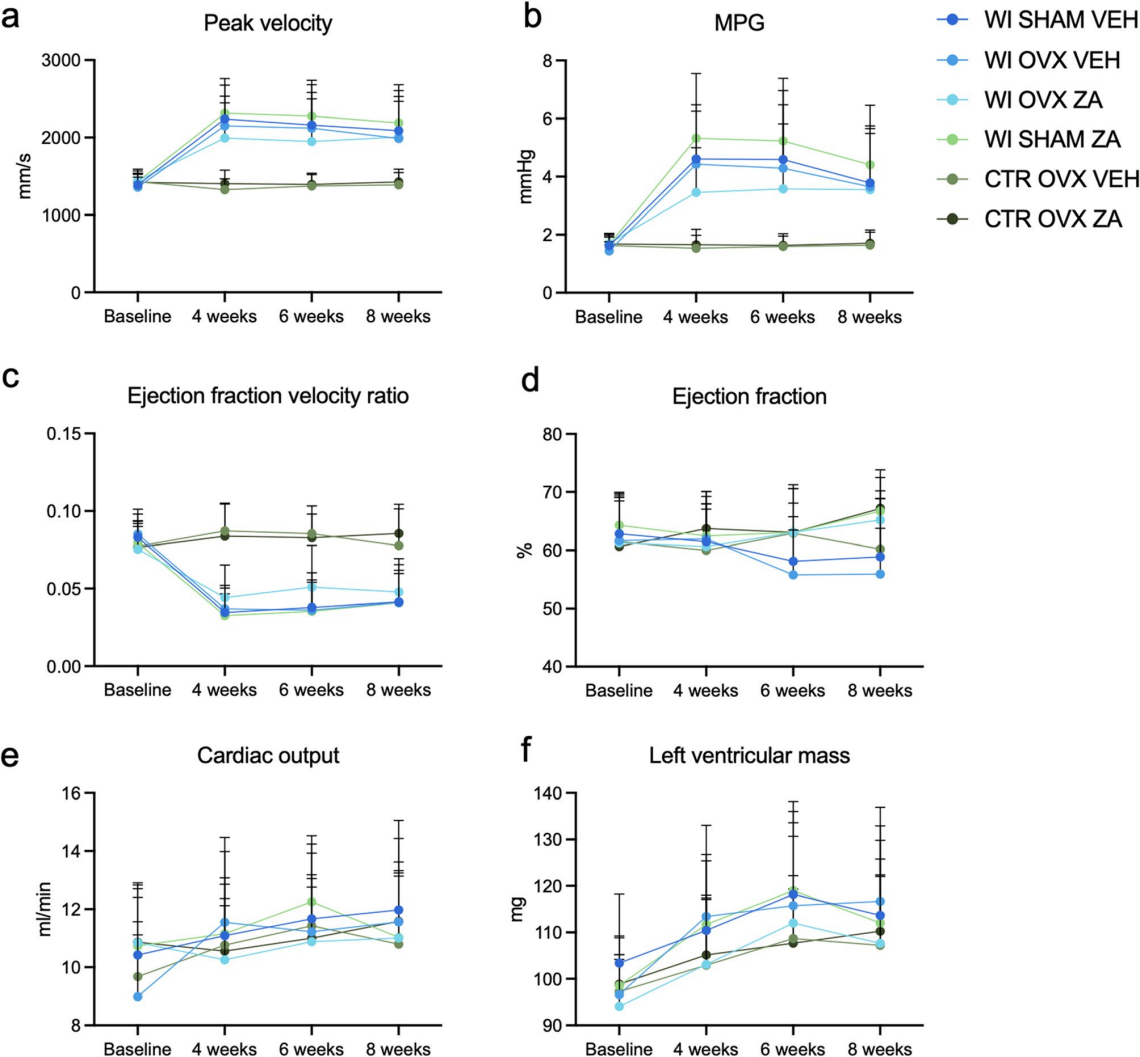
Echocardiographic assessment of aortic valve and cardiac function over time. **a, b** Peak velocity and mean pressure gradient (MPG) increased significantly after wire injury (WI) compared to control (CTR) groups, with no substantial differences between groups based on osteoporosis status or zoledronic acid (ZA) treatment. **c** Ejection fraction velocity ratio was unaffected by osteoporosis or ZA treatment across all groups. **d** Ejection fraction showed a trend toward increase in ZA-treated groups compared to vehicle (VEH)-treated groups. **e, f** Cardiac output and left ventricular mass remained stable across all groups during the 8-week observation period, indicating no overt cardiac dysfunction regardless of treatment or osteoporosis status

To examine the effect of osteoporosis on AVS, 2 weeks after OVX, AVS was induced via direct injury of the aortic valve with a coronary wire. Peak velocity at baseline was 1294 mm/s (SD 133.3 mm/s) and mean pressure gradient (MPG) was 1.58 mmHg (SD 0.34 mmHg). Wire injury (WI) successfully induced AVS in all groups, shown by a significant increase in peak velocity (*p* < 0.0001, Fig. 2a) and MPG (*p* < 0.0001, Fig. 2b) 2 weeks after the procedure (4 weeks after OVX). AVS remained hemodynamically stable until the end of the experiment (Fig. 2a, b).

Heart rate and blood pressure were measured every 2 weeks. OVX and WI did not influence these parameters (Supp. Mat. e-f).

### No effect on wire injury-induced aortic valve stenosis by ovariectomy or ZA

There were no differences in occurrence or progression of wire-injury induced AVS between groups with or without OVX-induced osteoporosis, as shown by similar peak velocity and MPG in groups after OVX/sham and ZA/vehicle (Fig. 2a, b). After control surgery, OVX alone or OVX and ZA did not influence peak velocity or MPG in comparison to baseline values (Fig. 2a, b).

Ejection fraction was 62.1% (SD 7.3%) at baseline and was not impaired by the manifestation of AVS or after OVX (Fig. 2d). Ejection Fraction Velocity Ratio (EFVR) was used to account for individual influences of ejection fraction on peak velocity. EFVR was significantly reduced following wire injury (Fig. 2c). However, OVX or ZA did not lead to any additional reduction in EFVR. After the initial post-wire injury decline, EFVR values remained stable throughout the study, showing no further significant changes at 6 or 8 weeks in any group.

Interestingly, ZA led to a slight increase of the ejection fraction in all groups and over time, whilst cardiac output was not affected by ZA injections.

### No effect on left ventricular mass by ovariectomy

Left ventricular (LV) mass was measured by echocardiography. Whilst at baseline, LV-mass was 98 mg (SD ± 11 mg), it increased over time with no clear differences related to interventional groups (Fig. 2f).

### Histological analysis

Histological analysis of the aortic valves was performed following animal euthanasia.

**Fig. 3:**
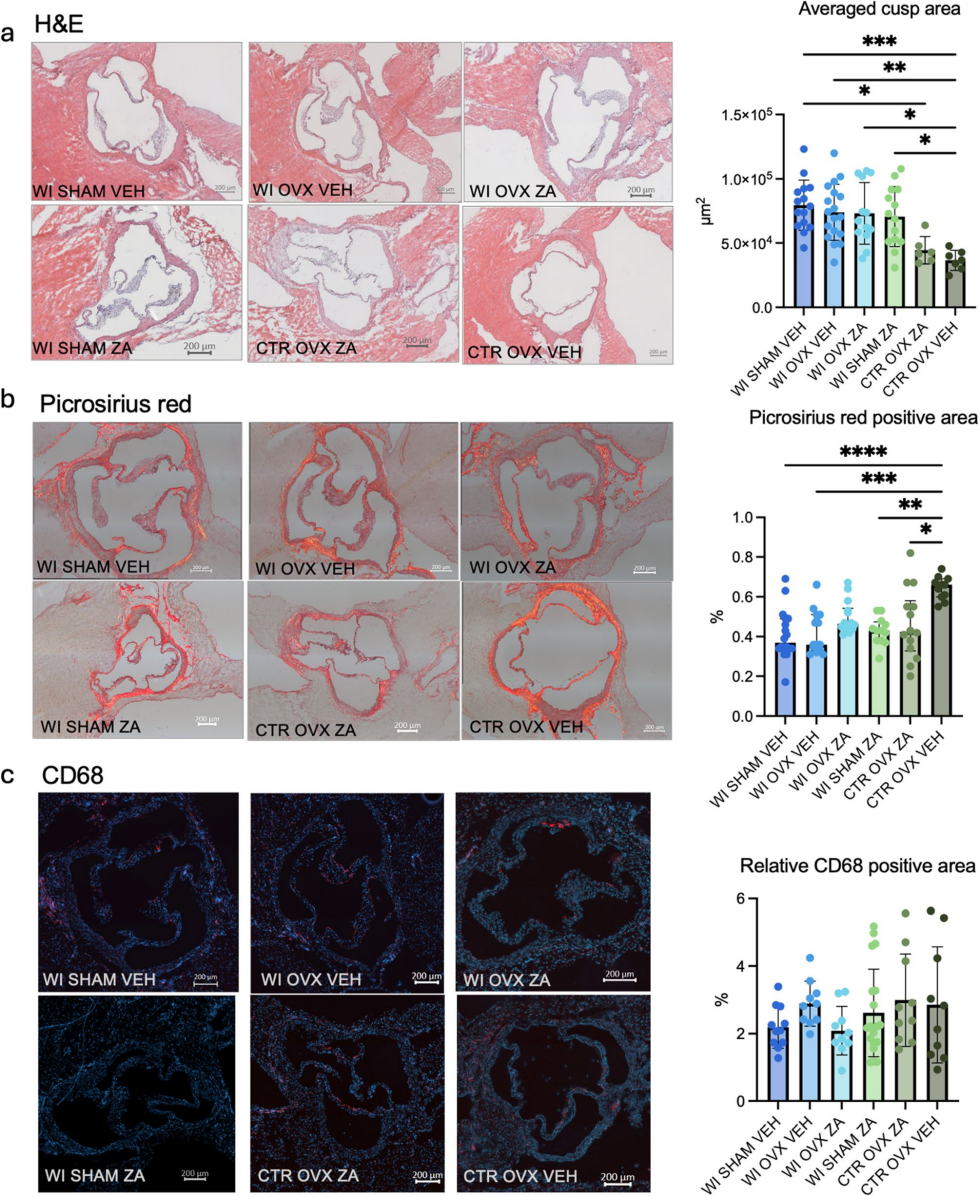
Histological and immunohistochemical analysis of aortic valves. **a** Hematoxylin and eosin (H&E) staining revealed increased cusp area in WI groups, with no significant differences based on ZA treatment or osteoporosis status. **b** Picrosirius red staining showed increased collagen deposition in WI groups, with higher levels observed in ZA-treated animals. **c** CD68 immunostaining indicated elevated immune cell infiltration in WI groups, independent of ZA treatment or osteoporosis status

Hematoxylin and eosin staining revealed a significantly enlarged cusp area in WI group compared to the control surgery groups, consistent with echocardiographic findings (Fig. 3a).

Collagen content was visualized using Picrosirius Red (PSR) staining. While the Group with OVX only (CTR OVX VEH) exhibited significantly increased collagen deposition compared to other groups, this observation was not consistent across other ovariectomized groups nor clearly associated with AVS status (Fig. 3b).

CD68 staining did not show an effect on immune cell infiltration within the aortic valves of animals subjected to WI, regardless of osteoporosis status (Fig. 3c).

No valvular calcification was detected in any group using Alizarin Red S and OsteoSense 680 EX staining (OsteoSense 680 EX images shown in Supplementary material, Alizarin Red staining not shown).

## Discussion

In this study, we aimed to explore the relationship between early-stage AVS and osteoporosis– two conditions that predominantly affect the elderly and are becoming increasingly prevalent with demographic changes.

The purpose of our animal model was to investigate the impact of osteoporosis, induced by bilateral ovariectomy (OVX), on the development and progression of AVS. The bisphosphonate ZA was administered to serve as a control for osteoporosis-related changes by preventing bone resorption.

To our knowledge, our study was the first to investigate the OVX-model of osteoporosis together with an animal model for AVS.

Within our OVX/WI mouse model, estrogen-deficiency–induced osteoporosis did not lead to early microcalcification, nor did it exacerbate early fibrotic thickening, collagen deposition or hemodynamic obstruction of the aortic valve six weeks after mechanical injury. Likewise, high-dose ZA, while completely reversing osteoporotic bone loss, did not alter these early valvular changes. These findings suggest that the epidemiological link between osteoporosis and advanced calcific AVS may arise at later, mineralization-dependent stages that were not captured within our 8-week observation window.

We observed a marked rise in collagen staining in CTR OVX VEH cusps, whereas collagen content in all wire-injured groups– including OVX and ZA-treated animals– remained comparable to sham controls. Estrogen deficiency is known to elicit chronic, low-grade, pro-fibrotic signaling [32, 33]. By contrast, wire injury triggers an acute inflammatory surge [26]. Mechanical endothelial damage, together pro-inflammatory cytokines released by infiltrating immune cells may therefore up-regulate MMP-2/-9 and accelerate extracellular-matrix turnover and collagen degradation [34].

Antifibrotic effects of ZA have likewise been reported by Komatsu et al. and others, who showed that the drug blocks TGF-β-induced myofibroblast differentiation and collagen synthesis in human gingival fibroblasts [35, 36].

Although epidemiological studies have highlighted an association between osteoporosis and cardiovascular disease, and preclinical research has identified shared pathways, our animal model suggests that bone loss alone may not directly aggravate wire injury-induced AVS. These findings are in line with a previous study from Joll et al. that investigated bilateral OVX combined with a Western diet as a model for left heart disease [37]. Over a one-year experimental period, ovariectomized mice exhibited a significant increase in left ventricular mass. Unlike our study, this group did not induce AVS through direct injury. Joll et al. reported no evidence of aortic valve hypertrophy, fibrosis, or calcification in their findings.

Hjortnaes et al. have identified a strong inverse correlation between vascular and valvular calcification and bone mass in ApoE^−/−^ mice. Whilst from epidemiological data it is known that osteoporosis and cardiovascular calcification share risk factors, including hyperlipidemia and inflammation, Hjortnaes et al. provided mechanistic, in vivo evidence linking these processes [38]. Although Hjortnaes et al. provided further experimental data for the “calcification paradox” in the context of ApoE deficiency-induced hyperlipidemia, they did not report the occurrence of hemodynamic relevant AVS in their study. Under standard conditions, ApoE^−/−^ mice are not known to develop hemodynamically significant AVS [39].

Calcium and phosphate levels in the serum are tightly regulated by hormones such as parathyroid hormones, calcitriol, and calcitonin, keeping them stable even during increased bone turnover [40]. That is why in our study, osteoporosis induction was controlled by µCT-imaging and histological analyses [41]. Given the epidemiological inverse correlation between valvular and vascular calcification with bone mass, some clinical retrospective and observational studies have even pointed towards bisphosphonate use being associated with lower progression rates of AVS [42, 43]. Bisphosphonates exert their anti-osteoporotic effects by inhibiting bone resorption through reducing the activity of osteoclasts. Their potential impact on AVS has been attributed to their ability to reduce inflammatory cytokines, suppress matrix metalloproteinase production, and halt bone resorption with its consecutive release of procalcific substances [5].

Based on the epidemiological and preclinical findings, Pawade et al. set up the double-blind, randomized, controlled trial SALTIRE [13] II that investigated the effects of the bone resorption inhibitors, denosumab and alendronate, on aortic valve calcification and AVS. No effect could be seen on the progression of aortic valve calcification or peak velocity after 2 years [13]. These negative findings might be a consequence of a serious impairment of bone resorptive activity in the cardiovascular system that has been well described for atherosclerotic lesions in the past [10, 44, 45].

It has also been suggested that the findings of SALTIRE II might reflect the hypothesis that the pathophysiology of AVS is independent of bone turnover [10]. In line with this is a study that found that denosumab, a RANKL inhibitor, did not affect aortic calcification and cardiovascular events over three years in postmenopausal women with osteoporosis [46].

Our finding that high-dose ZA failed to modify peak aortic velocity aligns with these clinical observations that potent anti-resorptives do not reverse valve obstruction. Because our model reflects an earlier, pre-calcific stage, direct comparison must be made with caution.

Abdoun et al. prospectively tracked 33 post-menopausal women with mild aortic stenosis for a median of 2 years and showed that those entering menopause early progressed more quickly– both in valve-calcium load and transvalvular gradient– whereas hormone-replacement therapy (HRT) tempered this trajectory [47]. Their cohort, however, represents the calcification-dominant stage of disease, while our eight-week model captures a fibrotic phase, so the absence of an OVX effect in our data does not contradict their findings. Moreover, the clinical study is limited by its small sample size, retrospective HRT reporting and potential residual confounding, and it did not measure bone-mineral density or skeletal markers.

Most former studies on models of AVS in mice have been conducted on males [26, 48–51]. Fleury et al. have evaluated the impact of sex and gonadal status on AVS in LDLr^−/−^ ApoB^100/100^ IGF- II^+/−^ mice with and without gonadectomy and under a high-fat/high-sucrose/high-cholesterol diet. Male mice, particularly those with intact testosterone levels, exhibited greater aortic valve calcification and faster AS progression after 36 weeks, while female mice were more resistant to calcification but developed fibrotic remodeling instead, independent of gonadal status [52]. Limited data exist on the applicability of the wire injury model for AVS in both sexes. Our study confirms that this model reliably induces AVS in female mice. Throughout the observation period, Alizarin Red and Osteosense staining did not reveal calcification, consistent with findings by Fleury et al. [52]. Our choice of female mice was driven by the association of postmenopausal osteoporosis and calcific AVS in human patients. Future studies could include male mice subjected to orchiectomy as well as extended follow-up periods to allow for the simultaneous evaluation of osteoporosis induction and calcific progression in this model.

Our model has several limitations that may restrict its applicability to human disease. First, the osteoporosis model employed in this study was based on a postmenopausal approach, which is commonly used in research settings [53, 54]. While postmenopausal osteoporosis represents the most prevalent form of the disease, it is important to acknowledge that osteoporosis can arise from various other causes, such as chronic glucocorticoid use, endocrine disorders, inflammatory conditions, and chronic kidney disease [55, 56]. Our study did not investigate these specific conditions or their potential interactions with osteoporosis in influencing AVS progression. To control for non-osteoporotic effects associated with OVX, ZA was applied to prevent bone resorption in animals after OVX or sham procedure.

Several murine models have been developed to mimic facets of AVS, each capturing only a subset of the clinical spectrum. The WI model used here generates a reliable, hemodynamically measurable gradient within ~ 2 weeks, making it well suited for intervention studies that require an early and reproducible rise in peak velocity. By contrast, genetic hyperlipidemic models (e.g. ApoE-/-, LDLr-/-) predominantly promote lipid deposition and histologically detectable leaflet thickening, but often produce inconsistent or late hemodynamic obstruction [39]. Future work should combine these complementary approaches to obtain a more complete picture of valve pathology.

Human AVS gradually develops over years, thought to be a result of prolonged exposure to cardiovascular risk factors (e.g., hyperlipidemia, blood flow alterations, inflammation) or as a result of genetic alterations, whereas the wire injury model induces a controlled, rapid mechanical damage, which limits extrapolation to the human setting, where most patients develop AVS in the absence of direct valve trauma and over much longer time-scales [57–61].

The absence of detectable (Alizarin Red S- and Osteosense 680 EX-staining) indicates that our model reflects pre-calcific remodeling.

Previous studies have shown that pro-inflammatory or pro-calcific stimuli were able to affect wire-injury induced AVS during a short period of six weeks after the procedure [27, 51]. However, macrocalcification in the wire injury model typically occurs only after 16 weeks [50, 62]. Our endpoint therefore interrogates inflammatory and fibro-calcific initiation rather than fully mineralized stenosis. While we anticipated that osteoporosis might accelerate calcification in early stages of AVS, neither Osteosense 680 EX imaging nor Alizarin Red S detected mineralization within this timeframe. Accordingly, our data do not refute the “calcification paradox” and cannot address whether osteoporosis or ZA influence late, mineralization-driven progression of AVS. Prolonged follow-up studies (≥ 16 weeks) or genetically engineered models that calcify earlier will be required to resolve this question.

Additionally, our study was limited by the inability to perform quantitative protein or transcriptomic analyses due to the restricted availability of murine aortic valve tissue samples.

## Conclusion

In summary, our study successfully induced osteoporosis in ovariectomized mice and demonstrated that ZA effectively preserved trabecular bone structures. Within the pre-calcific window studied, estrogen-deficiency-induced bone loss did not amplify early fibrotic thickening or hemodynamic obstruction. Whether osteoporosis accelerates the later calcific progression of AVS remains to be determined in longer-term models. However, additional studies are necessary to rule out the possibility that these results are influenced by the limitations of our study.

## Electronic supplementary material

Below is the link to the electronic supplementary material.

Supplementary Material 1

## Data Availability

The datasets used and/or analysed during the current study are available from the corresponding author on reasonable request.
