# Supplementary material for: Aortic valve stenosis and osteoporosis: insights from a mouse model
Source: BMC Cardiovasc Disord. 2025 Jul 31;25:562. doi: 10.1186/s12872-025-05037-4 (PMC12312273; doi:10.1186/s12872-025-05037-4)
Supplement: Supplementary file 1 — Supplementary Material 1 [file 12872_2025_5037_MOESM1_ESM.pdf]

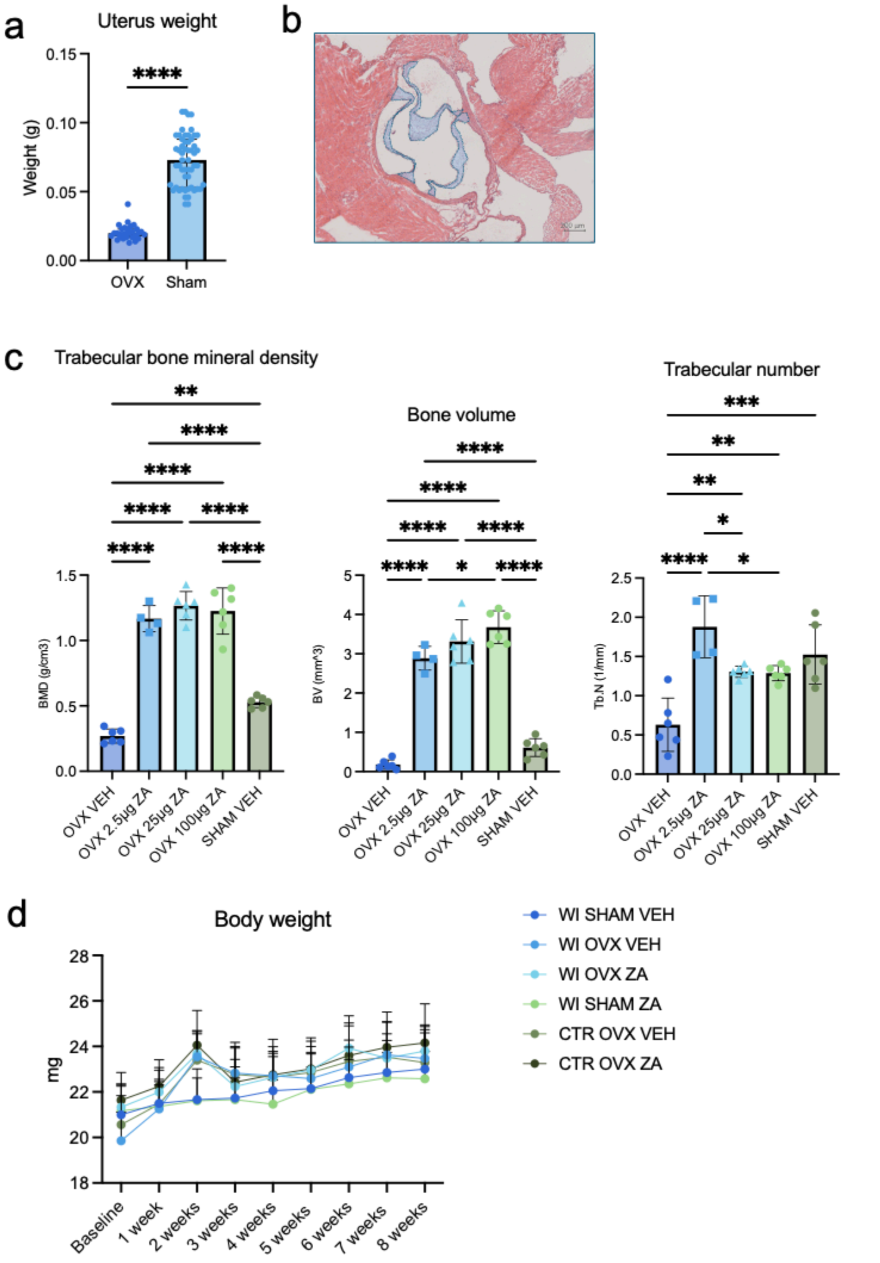

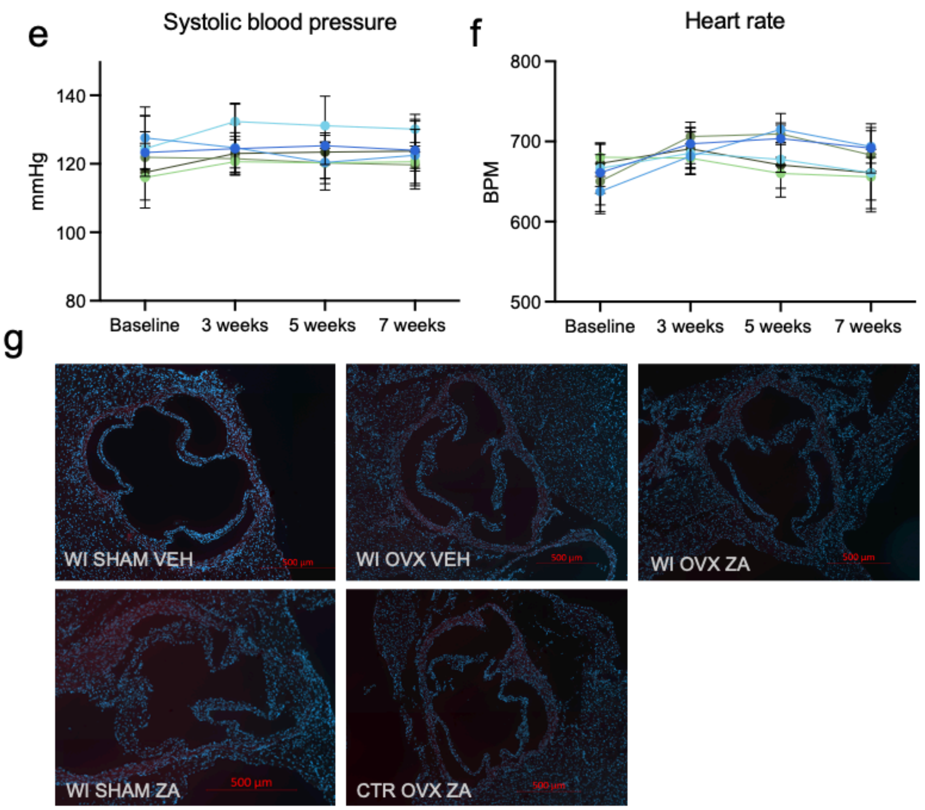

"Aortic Valve Stenosis and Osteoporosis: Insights from a Mouse Model", Heart and Vessels, Hannah Billig, Johanna Schmitt, Lamia Singer, Christoph Bourauel, Frank A. Schildberg, Werner Masson, Wenzel Vogel, Sven Perner, Marta Stei, Sven Niepmann, Miriam Silaschi, Farhad Bakhtiary, Georg Nickenig, Sebastian Zimmer.
hannah.billig@ukbonn.de, Department of Cardiology, University Hospital Bonn
